# A snapshot of the prevalence of physical activity amongst older, community dwelling people in Victoria, Australia: patterns across the 'young-old' and 'old-old'

**DOI:** 10.1186/1471-2318-7-4

**Published:** 2007-02-23

**Authors:** Jane Sims, Keith Hill, Sandra Davidson, Jane Gunn, Nancy Huang

**Affiliations:** 1 School of Physiotherapy, University of Melbourne, Melbourne, Australia; 2 National Ageing Research Institute, Melbourne, Australia; 3 Department of General Practice, University of Melbourne, Melbourne, Australia; 4 National Heart Foundation, Melbourne, Australia

## Abstract

**Background:**

Physical activity has a range of health benefits for older people. The aim of this study was to determine physical activity prevalence and attitudes amongst respondents to a trial screening survey.

**Methods:**

A cross-sectional survey was conducted. Subjects were community dwelling older people aged ≥ 65 years, recruited via general practices in Victoria, Australia. Participants completed a mailed screening tool containing the Geriatric Depression Scale, the Active Australia survey and the Physical Activity Readiness Questionnaire.

**Results:**

Of 330 participants, 20% were ≥ 80 years. Activity levels were similar to those reported in population studies. The proportion of participants reporting physical activity was greatest for the walking category, but decreased across categories of physical activity intensity. The oldest-old were represented at all physical activity intensity levels. Over half reported exercising at levels that, according to national criteria are, 'sufficient to attain health benefit'. A greater proportion of participants aged 85 years and older were unaware of key physical activity messages, compared to participants aged less than 85 years.

**Conclusion:**

Most population surveys do not provide details of older people across age categories. This survey provided information on the physical activity of people up to 91 years old. Physical activity promotion strategies should be tailored according to the individual's needs. A better understanding of the determinants of physical activity behaviour amongst older sub-groups is needed to tailor and target physical activity promotion strategies and programs to maximise physical activity related health outcomes for older people.

## Background

The WHO has emphasised the value of physical activity participation:

" Physical activity is the single most useful thing that individuals can do to maintain their health and function and quality of life" [1].

The estimated direct health care costs attributable to inactivity across the Australian population are about $AU377 million/annum [2]. Although physical inactivity is a problem across the lifespan, the older population accounts for a significant proportion of these costs. Older people are at increased risk for ill health and functional impairment due to sedentary lifestyle. Less than half of Australians aged 65 years and over do sufficient physical activity to produce health benefit (defined as 150 minutes or more of moderate and/or vigorous activity/week)[3]. Given the absolute risk of ill health associated with inactivity in older people, we have much to gain at an individual and societal level from older people being physically active.

There is ample evidence that lower mortality rates are associated with regular physical activity [4]. Physical inactivity is an independent risk factor for heart disease, accounting for 7% of the overall disease burden, second only to smoking (12%) [2,5]. Physical activity can ameliorate the onset, progression and severity of many chronic conditions, including reduced incidence of hypertension, heart disease, osteoporosis, degenerative arthritis, colonic cancer and diabetes mellitus, improved mood and memory function, and maintained social networks [6]. Regular physical activity is also associated with increased self-esteem and self confidence [7].

Older people may benefit from physical activity even more than other age groups. Talbot and colleagues [8] reported that the protective effect of leisure time activity for cardiovascular disease prevention is more important for older than for younger men. Further, systematic review evidence indicates that older people are more likely to participate in physical activity programs than younger adults [9]. Physical activity may offer a useful alternative to drug management and reduce the need for medication in some conditions, such as hypertension and Type 2 diabetes in older people [9].

Bauman et al. [10] compared national physical activity levels in Australian adults in 1997 and 1999 and reported a 6% decline in rates of those sufficiently active (63% to 57%). The decline was greater than that reported for other countries, such as the United Kingdom, New Zealand, the United States of America and the Netherlands [11,12]. At this time the national decline was not observed amongst older adults, although interpretation was limited by the sample's lack of differentiation amongst those aged 75 years and over. The most recent data from the 2004–2005 Australian National Health Survey confirms the prevailing trends reported in the literature [13]. Compared to the 2001 figures, the proportion of those 65 years and above who are sedentary has increased, particularly amongst those ≥ 75 years, where an increasing proportion is not sufficiently active to achieve health benefits. Walking for physical activity is most common (around 54%) in the younger old age groups, i.e. 55–64 and 65–74 years, but lowest in those aged 75 years and over (35.7%). Amongst women aged 75 years and over, the proportion categorised as engaged in moderate levels of physical activity decreased from 17% in 1995 to 11.6% in 2004–2005.

This paper reports on physical activity behaviour amongst community dwelling older people in Victoria. The data was obtained whilst screening people for inclusion in a trial exploring the use of strength training to manage depression in older people [14]. Studies of physical activity trends have chiefly focused on the 'younger old': relatively few sampling frames have included the 'old-old', i.e., those over 85 years. The current research aimed to address this gap in the evidence base by reporting on the physical activity patterns of older Victorians across a broad age range.

## Methods

### Subjects and setting

The study was conducted in the state of Victoria, Australia. General Practitioners (GPs), via Divisions of General Practice and phone listings, were invited to assist in recruiting older people for the study. Forty GPs from 85 practices agreed to participate in recruitment of eligible older patients. The trial's main aim was to attract people with depressive symptoms who were interested in participating in a strength training program, thus the trial eligibility criteria were: age 65 years or above and Geriatric Depression Scale (GDS) [15] score ≥ 11. For the research reported here, only age was used as an eligibility criterion.

Almost a thousand people (984) aged ≥ 65 years were recruited, primarily via invitation letters from participating practices distributed to all living patients aged ≥ 65 years. This recruitment strategy was supplemented by awareness raising campaigns at practices using information leaflets and posters and by opportunistic recruitment by GPs. Ethical approval for the study was obtained from the University of Melbourne's Human Research Ethics Committee. The Committee operates in accordance with the National Statement on Human Experimentation, based on the principles set out in the Helsinki Declaration.

### Measurements

People who agreed to participate completed a screening tool containing established questionnaires that, with the exception of the Active Australia Survey (AAS)[3], have been previously validated with older people:

1. The 30 item Geriatric Depression Scale (GDS-30) seeks a yes/no response to 30 statements about affective mood status[15]. A score of 11 or more is the clinical cut-off for depressive symptoms.

2. The Physical Activity Readiness Questionnaire, a screening questionnaire to determine suitability to undertake physical activity programs [16]. It has 100% sensitivity and 80% specificity for detecting physical activity contraindications, such as unstable angina [17].

3. The Active Australia Survey (AAS) asks about different intensities of activity, namely continuous walking (for at least 10 minutes) for recreation or active transport; vigorous gardening/heavy work and vigorous and moderate physical activity [3]. The items relate to frequency (number of sessions) and duration (time spent) undertaking physical activity in the last week. Algorithms are used to classify respondents as 'sufficiently physically active to attain health benefit' (based on accumulating 150 minutes or more of at least moderate intensity activity/week). Views on statements about physical activity and health are also elicited. Although not specifically psychometrically tested with older people, the AAS has good criterion validity and reliability for Australian adults [18,19].

Participants completed the screening instruments at home at a time convenient to them and mailed the questionnaires back to the researchers using a reply paid envelope.

### Statistical analyses

Descriptive statistics were used to assess the rate, type and duration of physical activity. Chi squared tests, linear and logistic regression analyses were used to assess age and gender differences. Age was treated as a categorical variable (65–79, 70–74, 75–79, 80–84 and 85 years and over respectively).

## Results

Nine hundred and eighty four people were invited to participate. Three hundred and forty six screening instruments were returned (response rate 35%). Usable data was available for 330 people. The proportion of people in the study aged over 80 years (20%) and the proportion of women (64%) was higher than in the general population where 10% of the over 65's are 85 or over and 56% are female) [20] (Table 1). The overall proportion of current smokers (3%) was also lower than reported in national datasets (10.9%). The proportion (22%) of respondents who met the GDS threshold for depressive symptoms was comparable with that of the general population aged 65 years or over [21]. PAR-Q scores indicated that this was a reasonably healthy sample, although based on their PAR-Q score one third of respondents would require medical review before undertaking physical activity. Around one quarter (23%) had experienced heart trouble and an equivalent proportion joint or bone conditions (18% of the Australian population ≥ 65 years report heart, stroke or vascular disease and 49% arthritis [13]). Over half (57%) were unaccustomed to vigorous physical activity.

**Figure 1:**
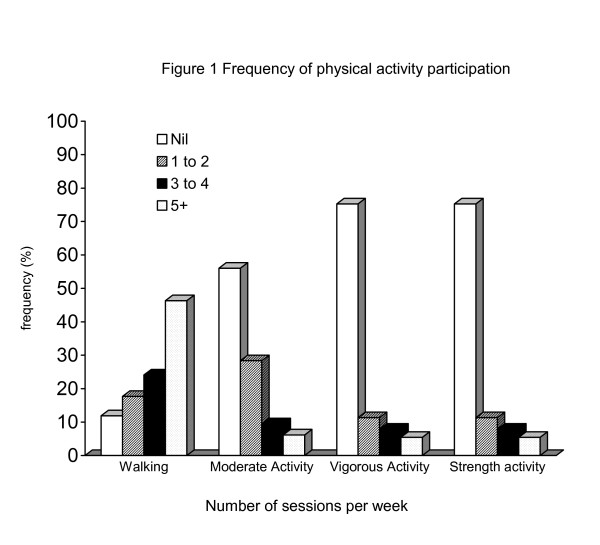
Frequency of physical activity participation.

Physical activity behaviour patterns were compared across gender and age groups. The findings are given in Figures 1 and Tables 2 to 4.

### Physical Activity Behaviour

Walking was the commonest physical activity mode, with more than half the respondents reporting three or more sessions per week (Figure 1). On average, respondents walked 146 minutes/week (SD 160). Women tended to walk fewer sessions, but for longer than men (mean 152.6 vs. 134.2 minutes), but the gender difference was not significant (p = 0.30 95% CI -54.9, 19.6, for difference) (Table 2). Men spent more time in moderate activity (mean 117 versus 78 minutes), but this was not significant (p = 0.17, 95% CI -16.54, 93.3, for gender difference). The majority did not engage in vigorous physical activity and there were no gender differences. As anticipated, rates of heavy work such as gardening, were higher in males (p = 0.008), with men spending more time on such tasks (153 vs. 55 minutes, p = 0.003, 95% CI 33.2, 163.1 for gender difference) (Table 2). The majority (85.7%) of respondents were not engaged in strength training type activities.

Based on AAS and national guideline criteria, 43.3% of the sample were sedentary, or at least not doing 'sufficient exercise to attain health benefit' at the time of completing the questionnaire (Table 3). There were no age or gender differences.

### Agreement with Physical Activity Messages

The respondents' agreement with physical activity behaviour messages was recorded. There was high agreement with population health messages (Table 4). Respondents largely agreed with the National Physical Activity guidelines message that incremental, moderate activity is acceptable. In turn, 50% disagreed with message three (that vigorous activity for at least 20 minutes at a time, three times a week is necessary to achieve health benefits). However, there was no statistically significant association between agreement with any of the health messages and reported physical activity behaviour, categorized according to minutes of overall activity (Spearman's rho was 0.09, 0.04, -0.11, -0.05 and 0.08 for messages 1 to 5 respectively with physical activity behaviour, p's > 0.01). There was a difference across age groups in agreement with two of the messages: 'Taking the stairs at work or generally being more active for at least 30 minutes each day is enough to improve your health' and 'Half an hour of brisk walking on most days is enough to improve your health', with those in the oldest age group being least likely to agree with these messages. This has implications for the impact of the current physical activity recommendations amongst the old-old group. These messages embody the principles of the National Physical Activity recommendations yet appear not to be affirmed by this section of the population.

### The Old-Old

Physical activity patterns were similar across age groups. Twenty percent of the sample was aged over 80. Of the 80–84 year olds 56.5% achieved sufficient physical activity/week for health benefits. For the 85+ group, the figure was slightly lower (44%). There was no difference in walking behaviour with age. Fifty three per cent of those 85+ did five or more sessions per week. Whilst the majority of the oldest age group reported no moderate activity sessions (81%), moderate physical activity duration did not significantly differ by age, due to the similar activity patterns amongst those who did participate in moderate activity. There was no age difference for vigorous physical activity: 75% of the 85+ group did none, compared to 71% of the 65–69 year olds. Although the oldest age group (85 + years) were not likely to participate in moderate and vigorous activity, there was no significant difference across age groups. The oldest age group also did the least amount of heavy work, but again this finding was not significant. The majority (86.4%) did not participate in any strength promoting activities. Most common types of physical activity for those aged over 80 included bowling, golf, playing with grandchildren and gardening.

## Discussion and Conclusion

The results of this study highlight that physical activity options sufficient to achieve health benefits are reported to be undertaken by approximately half of community-dwelling over 65 year olds recruited through general practice. Physical activity options, even some more vigorous approaches, appear to be acceptable to older people across all age groups. The findings can be used to guide strategies for engaging all sections of the older population in physical activity behaviour.

The findings are similar to others reported in the literature. For example, Brownie surveyed a random proportionate sample of over a thousand older Australians and found that 26% fell into the 'no activity' category and just over half into the 'high activity category' (at least 30 minutes five or more times per week) [22]. The study had limitations: intensity levels were not recorded, and housework and gardening were excluded from the categorisation of activity frequency/duration. The latest Exercise, Recreation and Sport Survey (ERASS), conducted by the Australian Sports Commission, indicates that the majority of respondents aged 65 years and over were involved in physical activity, particularly or non-organised physical activity (71.6%) [23]. Participation rates (any form of physical activity) decreased with age across the sample (from 91.7% in 15 to 24 year olds to 71.6% in over 65 year olds). Older people participated in fewer types of activity (1.6 different types on average): as in the current study, walking was the commonest form of activity (47.6% of older respondents).

In the current sample of people who responded to an invitation to participate in a physical activity trial, almost half were currently sedentary, or their level of exercise insufficient to obtain a health benefit. The evidence also indicates that agreement with physical activity messages does not translate into actual behaviour. These findings indicate that there is scope to further encourage physical activity in sedentary older people. Systematic reviews confirm that sedentary older adults can be encouraged to increase their physical activity [24]. The decision to complete the screening instrument suggests that many sedentary respondents were in 'contemplative' mode and that a health professional could work with them to promote physical activity behaviour. Trials have shown that brief advice given in primary care can promote behaviour change [25-27], but the level of intervention within primary care needed to encourage sustained physical activity in older people is unclear [28]. There is clearly a need for greater attention to physical activity promotion for older people in everyday general practice, such as via the use of the Australian Lifescripts program [29].

Various types of physical activity are useful for older people. Avenues to support greater levels of engagement of older people, including those over 80 years, in safe physical activity need to be explored. Strategies to optimize the accessibility and convenience of adopting physical activity have been shown to influence subsequent behaviour in children and young adults, but research with older people has been limited [9,28,30]. A recent review found that home-based, group-based and educational interventions can all increase physical activity, but the changes were modest and short-lived [9]. It is likely that a range of options, tailored to the individual, will be needed to assist the maintenance of physical activity behaviour. A range of models are currently being field tested in the USA as part of the National Blueprint program [31].

Given the variable agreement with the physical activity messages amongst the oldest age group in the present study, it seems pertinent to build a behavioural approach into any intervention seeking to encourage the old-old to become physically active. Relatively few intervention studies have focused on the old-old [32]. Simons and Andel [33] trialled two types of supervised physical activity in people with a mean age of 83.5 years. They found similar levels of improvement in functional status (albeit with no between group analyses) amongst those who followed a walking program and those completing a progressive resistance training program. For some 'frail' older people, walking may be a more feasible option in the long term, although they may require initial lower limb strength training to enable independent walking.

Presence of co-morbid conditions such as arthritis are often erroneously considered by older people (and others) to limit the ability to participate in *any *physical activity. Randomised controlled trials have identified that significant health benefits can be achieved by physical activity programs tailored for people with arthritis and other chronic conditions [34]. There is also established evidence that 'frail' older people can do strength training and benefit from it [35].

Interpretation of the study findings is limited by the recruitment mode. We recruited and surveyed participants from general practices in Victoria. It was thus not a random, population based sampling frame; recruitment depended upon GPs' agreement to assist. Nevertheless, with the exception of the proportion of old-old in our sample, the respondents were broadly representative of the older Australian population. Further, there are response bias problems associated with self-report of physical activity and agreement with physical activity messages [25,36,37], with over-estimation and social desirable responses potentially occurring. It may be that actual activity levels were insufficient to promote health benefits. We acknowledge that the prevalence figures are higher than those from multipurpose national surveys because they relate to the physical activity patterns of those responding to a specific physical activity survey, who are unlikely to be representative of the overall older population. More active older people are more likely to respond to both national and local physical activity surveys, influencing the findings. The sample is more likely to include those with an interest in physical activity than a true population sample.

With an ageing population, it is vital to support and maintain physical activity in older people to promote mental and physical health. Although a lifespan, population health approach is helpful, health policy and planning should be tailored to address the particular needs of older people. We need to know more about the physical activity determinants for specific subgroups of older people, such as the old-old. The Australian Government recently commissioned a project to produce physical activity recommendations for older people. When these are finalized and promoted widely, they should be useful in supporting increased physical activity among older people, including those with a range of co-morbidities and including those in the old-old age group.

## Competing interests

The author(s) declare that they have no competing interests.

## Authors' contributions

JS conceived and designed the study, obtained funding, oversaw subject recruitment and data analysis and prepared the paper. All authors contributed to the study design, project planning and preparation of the paper. All authors read and approved the final manuscript.

## Appendix

Sedentary: Less than 100 minutes of physical activity (ABS 2005)

Vigorous activity: based on physical activity for sport, recreation or fitness which caused a large increase in heart rate or breathing (ABS 2005)

Moderate activity: based on physical activity for sport, recreation or fitness which caused a moderate increase in heart rate or breathing (ABS 2005)

Sufficient: the accumulation of at least 150 minutes of activity over 1 week; or the accumulation of at least 150 minutes of activity and at least 5 sessions of activity over 1 week (Active Australia Survey).

## Pre-publication history

The pre-publication history for this paper can be accessed here:



## Figures and Tables

**Table 1:** Demographic characteristics of respondents (N = 330)

**Characteristic**	Number*	**Study sample (%)**	**Australian population (%)**
**Age **(years)			
65–69	74	27.5	12^a^
70–74	83	30.9	
75–79	59	21.9	
80–84	36	13.4	
85–91	17	6.3	10^b^
**Gender**			
Male	104	35.9	
Female	190	64.1	56
**Ethnicity**			
Australian born	167	61.9	68
Overseas born	103	38.1	
**Marital Status**			
Never married/single	8	3.0	
Married	159	59.8	76.4% men, 46.4% women^c^
Defacto/living with partner	5	1.9	
Separated but not divorced	8	3.0	
Divorced	14	5.3	
Widowed	72	23.9	
**Currently Smoking**	8	3.0	10.9^d^

* Numbers do not reflect the overall sample size due to mssing data across these variables

Source ABS 2003 [20] a Prevalence of over 65 year olds, b 10% of over 65's are 85 or over c percentages based on ' living with a partner' d 10.9% 65–74 year olds, 5.9% of 75 and over

**Table 2:** Duration of physical activity types (minutes)

**Gender**		**Walking**	**Moderate Activity**	**Vigorous Gardening/Heavy work**	**Vigorous Activity**
**Men**	Mean (SD)	134.2 (141.6)	117.6 (221.9)	*134.5 (256.6)	30.2 (75.6)
	Median	120.0	0	30	0
**Women**	Mean (SD)	152.6 (169.2)	85.3 (214.4)	59.2 (99.6)	36.5 (106.2)
	Median	120.0	0	0	0
**All**	Mean (SD)	145.9 (159.7)	96.9 (217.2)	86.5 (176.8)	34.2 (96.3)
	Median	120.00	0	10	0

* gender difference p < 0.05

**Table 3:** Proportions doing sufficient physical activity for health benefits (percentages)

**Category**	**Men**	**Women**	**All**	**65–69 years (n = 63)**	**70–74 years (n = 63)**	**75–79 years (n = 49)**	**80–84 years (n = 23)**	**85+ years (n = 9)**
No exercise	6.5	8.4	7.7	7.9	7.9	6.0	8.7	11.1
Less than 150 minutes per week	32.5	37.4	35.6	38.1	41.3	24.0	34.8	44.4
150 minutes or more per week	61.0	54.2	56.7	54.0	50.8	70.0	56.5	44.4*

* due to rounding, column percentages do not add to 100%

**Table 4:** Agreement with Physical Activity Messages (number and percent agreeing with the message)

**Message**	**Men**	**Women**	**All**	**65–69 years**	**70–74 years**	**75–79 years**	**80–84 years**	**85+ years**
1	76 (85.4)	133 (78.7)	209 (81.0)	64 (90.1)	67 (82.7)	45 (77.6)	24 (77.4)	9 * (52.9)
2	74 (81.3)	143 (84.6)	217 (83.5)	66 (90.4)	72 (86.7)	43 (74.1)	27 (90.0)	9 * (56.3)
3	58 (62.4)	88 (53.7)	146 (56.8)	46 (63.9)	46 (57.5)	31 (53.4)	16 (53.3)	6 (37.5)
4	64 (69.6)	131 (77.5)	195 (74.7)	52 (70.3)	66 (79.5)	42 (73.7)	23 (76.7)	12 (75.0)
5	83 (90.3)	160 (95.3)	243 (93.5)	69 (94.5)	78 (95.1)	53 (93.0)	28 (90.3)	15 (93.8)

Key:

Message 1 Taking the stairs at work or generally being more active for at least 30 minutes each day is enough to improve your health

Message 2 Half an hour of brisk walking on most days is enough to improve your health

Message 3 To improve your health it is essential for you to do vigorous exercise for at least 20 minutes each time, three times a week

Message 4 Exercise doesn't have to be done all at one time- blocks of 10 minutes are okay

Message 5 Moderate exercise that increases your heart rate slightly can improve your health

* p < 0.01
